# Supplementary figures and images for: Patterns of interactions among ICU interprofessional teams: A prospective patient-shift-level survey approach
Source: PLoS One. 2024 Apr 16;19(4):e0298586. doi: 10.1371/journal.pone.0298586 (PMC11020828; doi:10.1371/journal.pone.0298586)

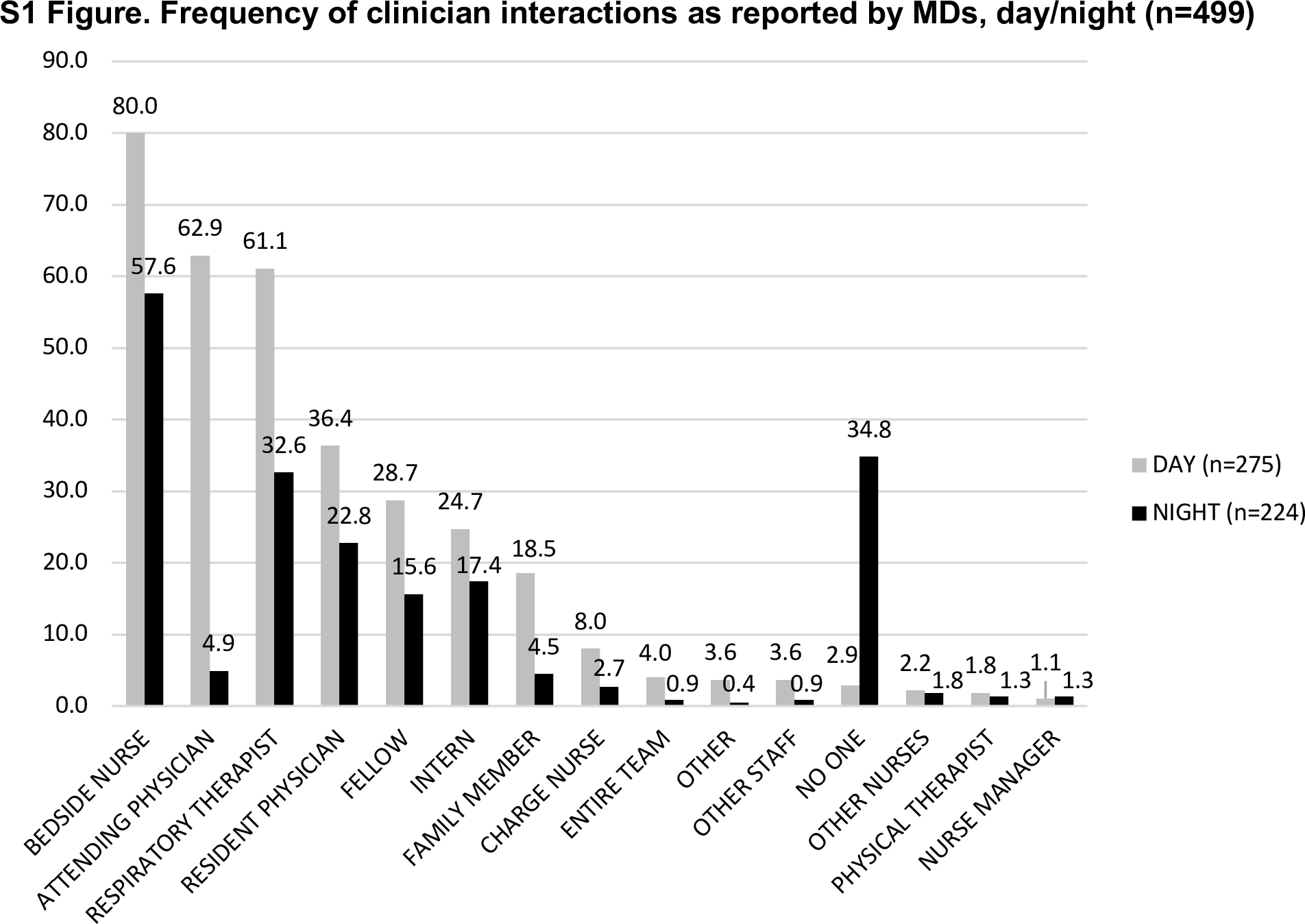

Supplement: S1 Fig — (TIF) [file pone.0298586.s001.tif]

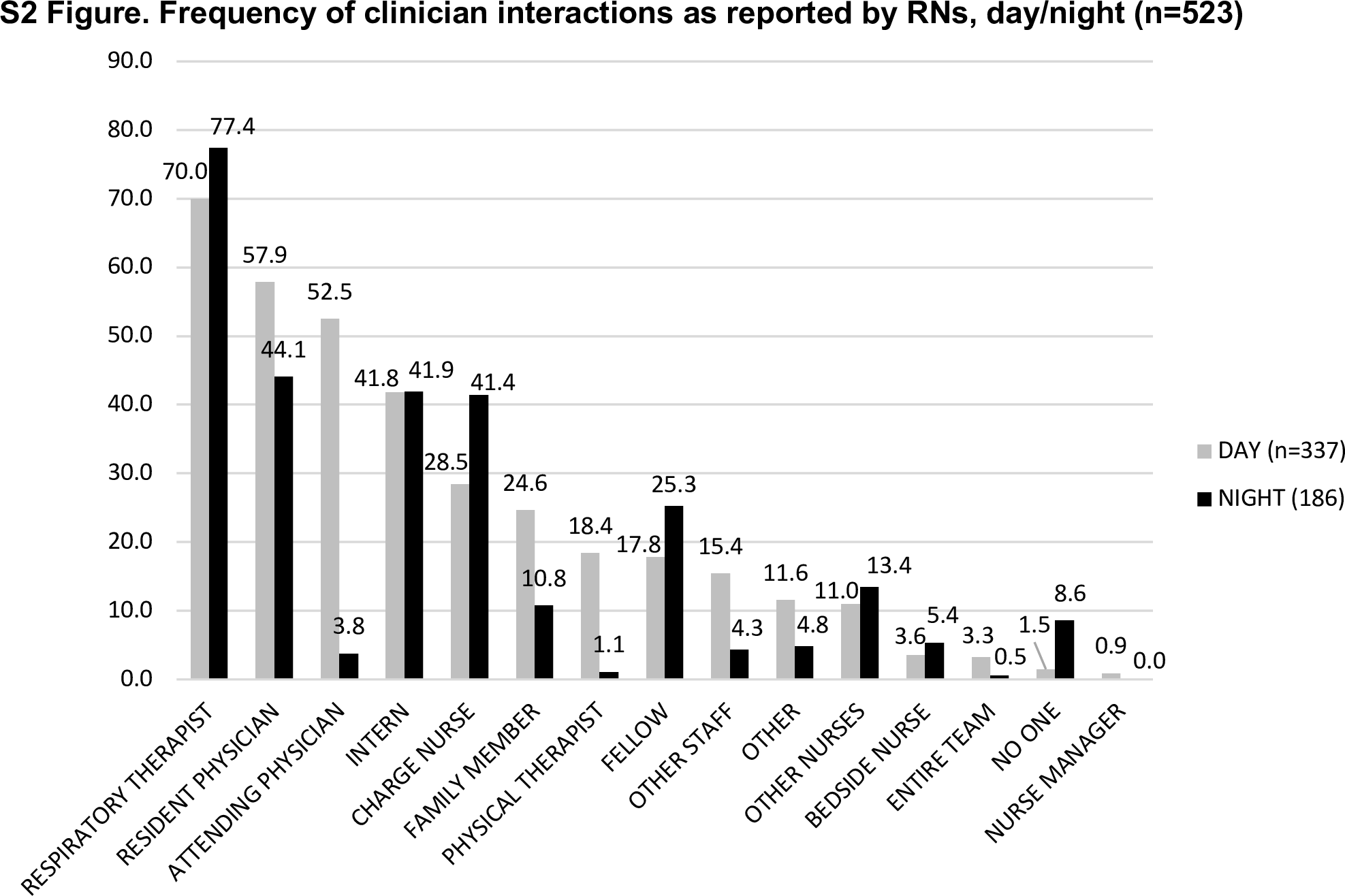

Supplement: S2 Fig — (TIF) [file pone.0298586.s002.tif]

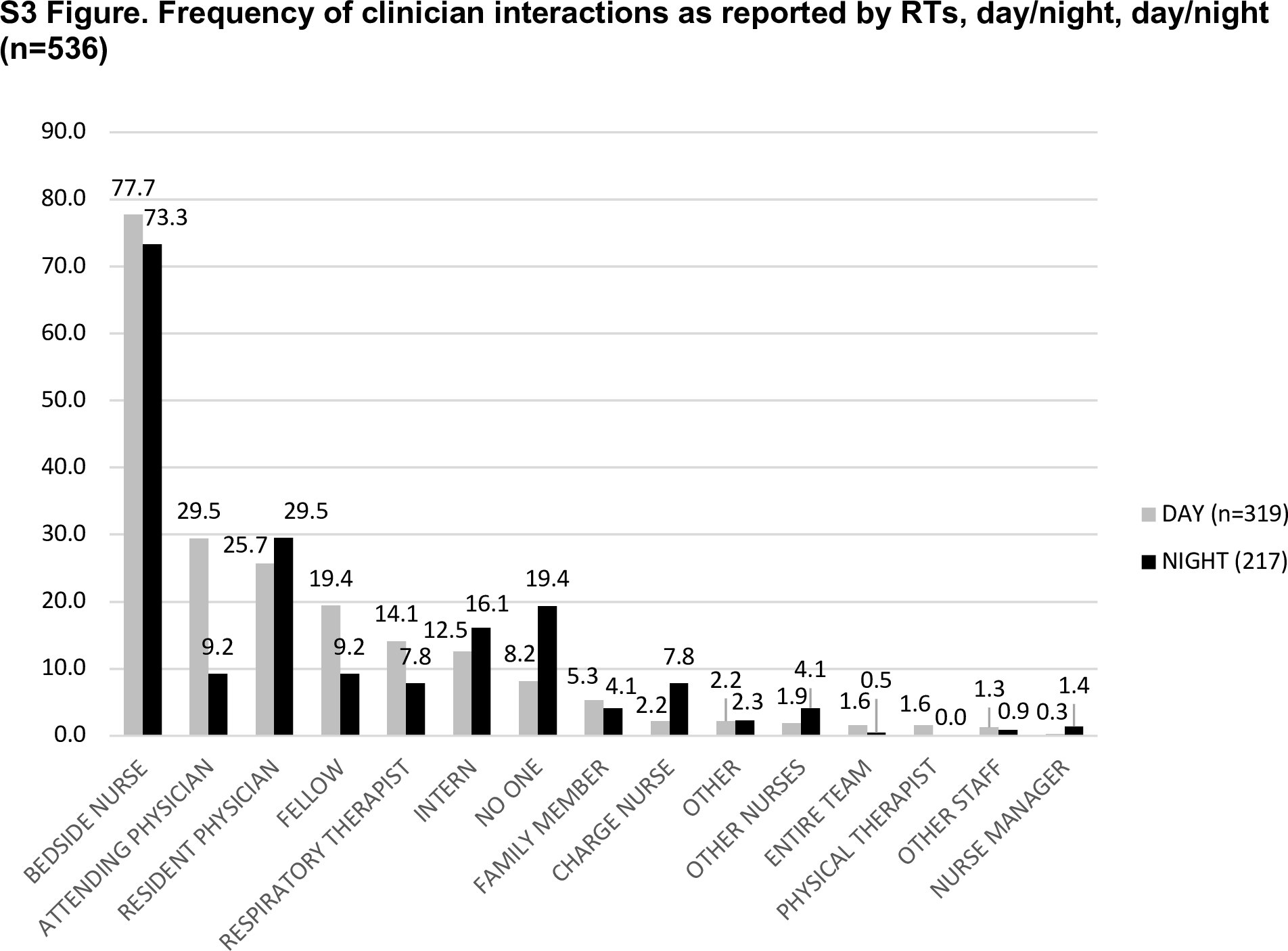

Supplement: S3 Fig — (TIF) [file pone.0298586.s003.tif]
