# Supplementary material for: Patterns of interactions among ICU interprofessional teams: A prospective patient-shift-level survey approach
Source: PLoS One. 2024 Apr 16;19(4):e0298586. doi: 10.1371/journal.pone.0298586 (PMC11020828; doi:10.1371/journal.pone.0298586)
Supplement: S1 File — (DOCX) [file pone.0298586.s004.docx]

Please refer to the care provided to your patient within the ABCDE bundle. Components of the bundle include: spontaneous awakening trials (or sedation vacations), spontaneous breathing trials, delirium assessment and early mobility. Please check all individuals and write in their names.

- Please check as many individuals as applicable and write in their names if you know their names
- Do not check yourself if your role is identified below
- Please report only contact that you had this shift, not contact others may have had on your behalf. We are not evaluating your care in any way.

**1. People you contacted this shift**

Who did **you contact** about ABCDE when caring for this specific mechanically ventilated patient?

(Contact could include communication about ABCDE during rounds, if you contacted the RT to discuss the spontaneous breathing tiral or if you contacted another nurse about the SAT, for example)

- Attending Physician:
- Resident Physician:
- Fellow: __________________________________
- Intern: __________________________________
- Respiratory Therapist:
- Physical Therapist:
- Bedside nurse:
- Charge Nurse:
- Nurse Manager:
- Other Nurses:
- Other Staff (fill in):
- Patient’s Family Member:
- Entire ICU team during morning rounds
- No one
- Other: __________________________________

**2. People you contacted for help this shift**

Who did **you** **contact** **for help** when problems arose with ABCDE for this patient?

- Attending Physician:
- Resident Physician:
- Fellow: __________________________________
- Intern: __________________________________
- Respiratory Therapist:
- Physical Therapist:
- Bedside nurse: ___________________________
- Charge Nurse:
- Nurse Manager:
- Other Nurses:
- Other Staff (fill in):
- Patient’s Family Member:
- Entire ICU team during morning rounds
- No one
- Other: __________________________________

**3. People who contacted you this shift**

Which individuals **contacted you** about ABCDE when caring for this specific mechanically ventilated patient?

- Attending Physician:
- Resident Physician:
- Fellow: __________________________________
- Intern: __________________________________
- Respiratory Therapist:
- Physical Therapist:
- Bedside nurse:
- Charge Nurse:
- Nurse Manager:
- Other Nurses:
- Other Staff (fill in):
- Patient’s Family Member:
- Entire ICU team during morning rounds
- No one
- Other: __________________________________

Thank you for your time spent taking this survey.
