# Supplementary material for: Patterns of interactions among ICU interprofessional teams: A prospective patient-shift-level survey approach
Source: PLoS One. 2024 Apr 16;19(4):e0298586. doi: 10.1371/journal.pone.0298586 (PMC11020828; doi:10.1371/journal.pone.0298586)
Supplement: Attachment — Submitted filename: Decision letter. docx.docx [file pone.0298586.s005.docx]

Dear Deena Kelly Costa,

Thank you for submitting your manuscript to Plos One Journal.

Based on Reviewers’ comments and my own assessment of your manuscript, I would like to inform you that your work doesn’t meet the criteria for publication with Plos One Journal in its current form, however, if you can address all reviewers concerns and my own comments, I can consider reviewing your revised manuscript.

Specifically, the manuscript needs to be re-written to clearly state the primary objective of the study and the main results in a consistent and non-confusing way. For example, in the abstract and results section, you state that the interaction with family members is common while it is below 20% for all 3 healthcare professionals. Please correct this unless if the standard of care is not to involve family members every time (100% of the time you do ABCDE).

In the methods section, you need to clearly define the standards of care or expected/ideal number of interactions between clinicians every shift? Also, comment on the ideal number of staff in your settings.

In your discussion, please explain if there is evidence that more interactions are associated with better care or better outcomes.

Also, make a comment about having less interactions during the night is expected or not, especially that some elements of ABCDE are not planned during the night like awakening and mobility because it is the time for sleeping for patients as well.

I am looking forward to reviewing your revised manuscript.

Yours sincerely,

Eugene Tuyishime, MBBS, MMed, MSc

Academic Editor

PLOS ONE
